# Supplementary figures and images for: Experimental methodologies can affect pathogenicity of Batrachochytrium salamandrivorans infections
Source: PLoS One. 2020 Sep 11;15(9):e0235370. doi: 10.1371/journal.pone.0235370 (PMC7485798; doi:10.1371/journal.pone.0235370)

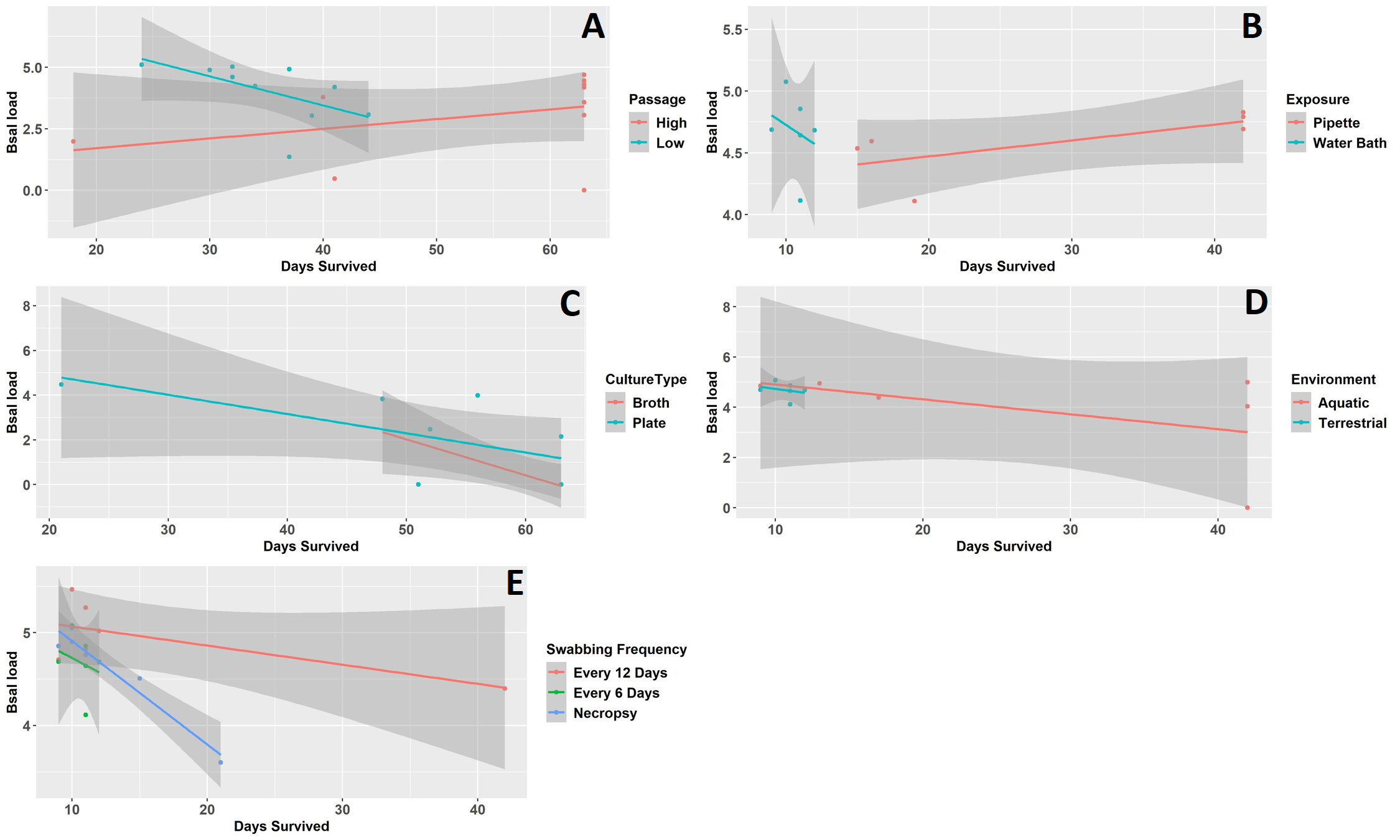

Supplement: S1 Fig — (TIF) [file pone.0235370.s001.tif]

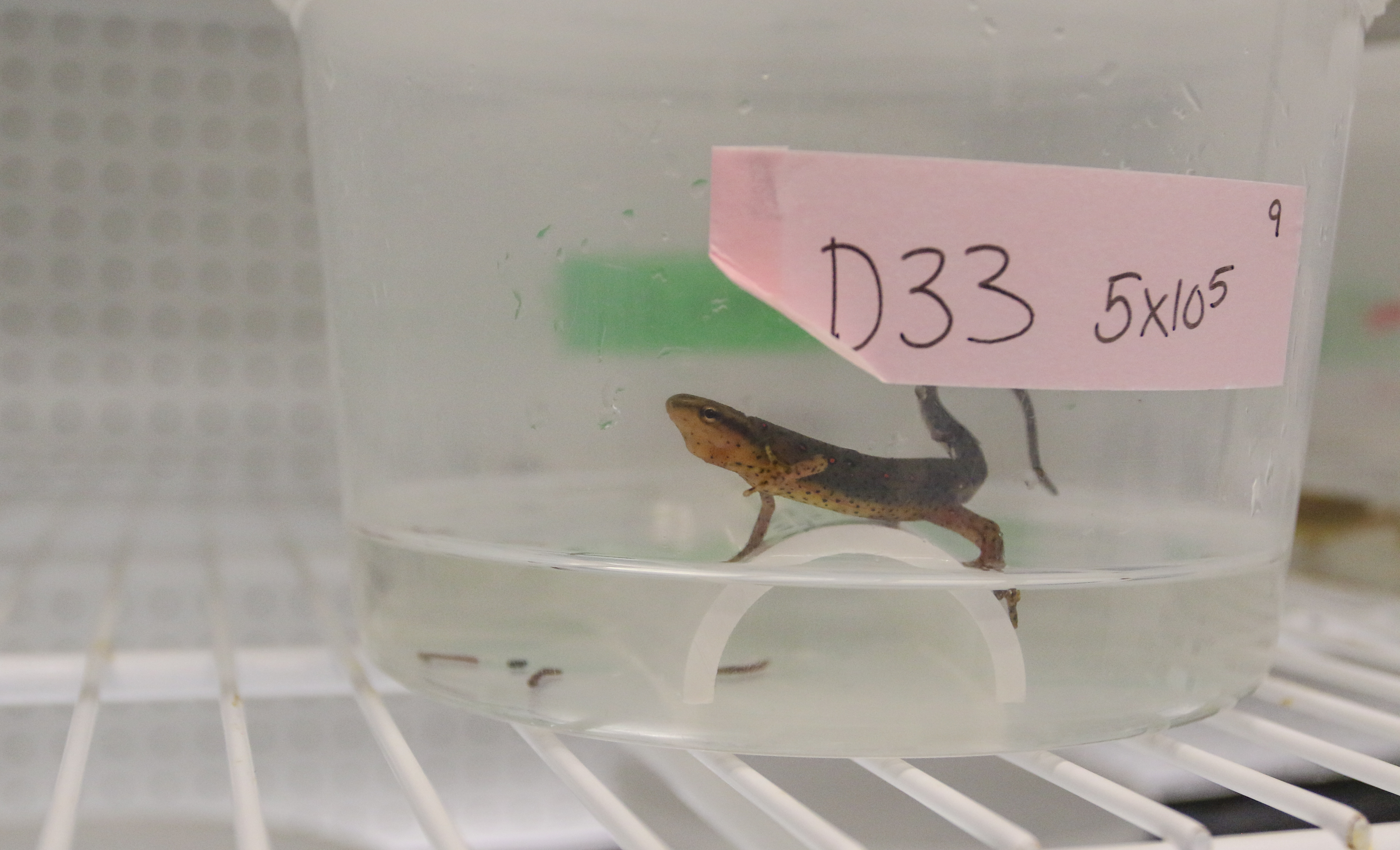

Supplement: S2 Fig — (JPG) [file pone.0235370.s002.JPG]

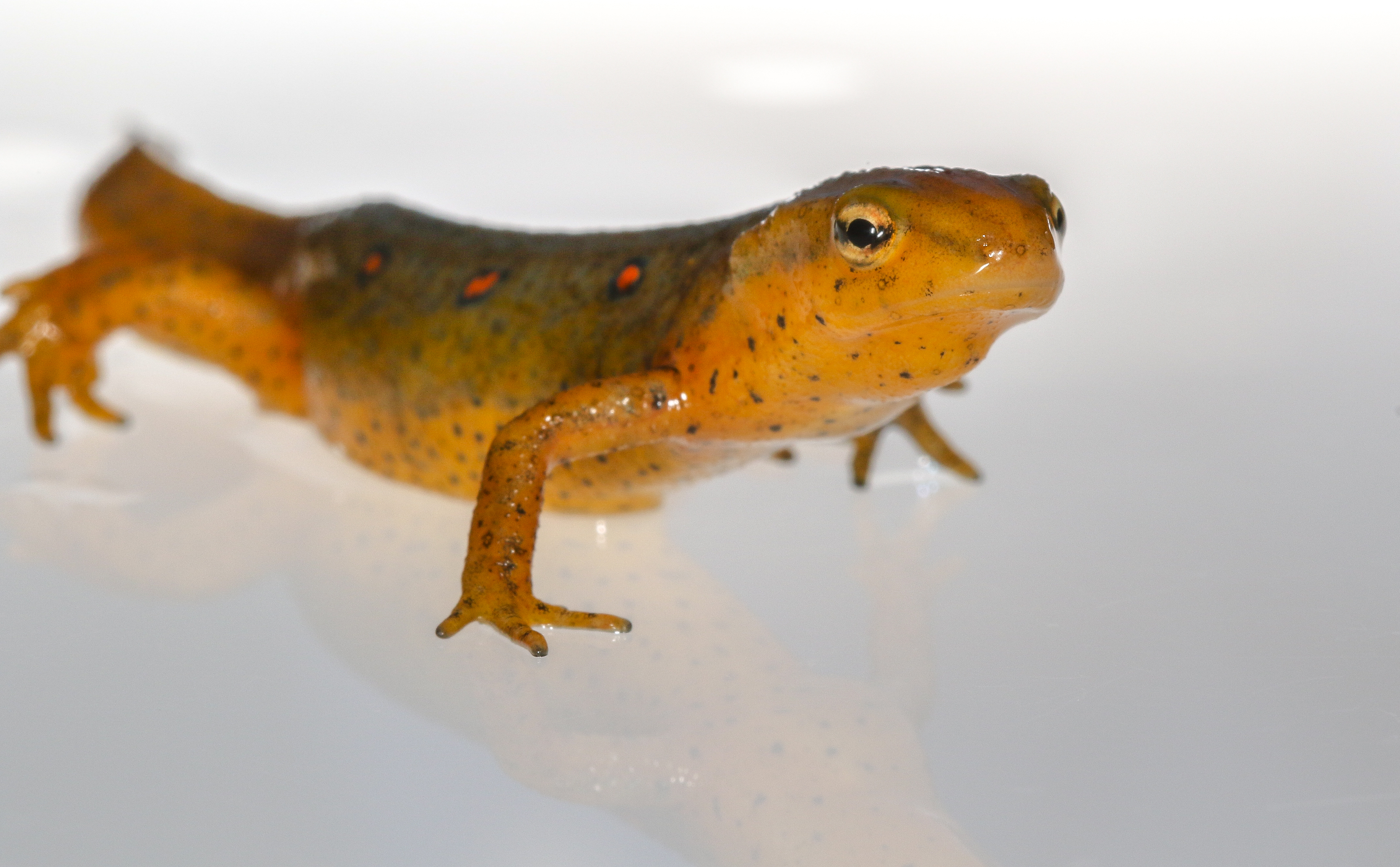

Supplement: S3 Fig — (JPG) [file pone.0235370.s003.JPG]

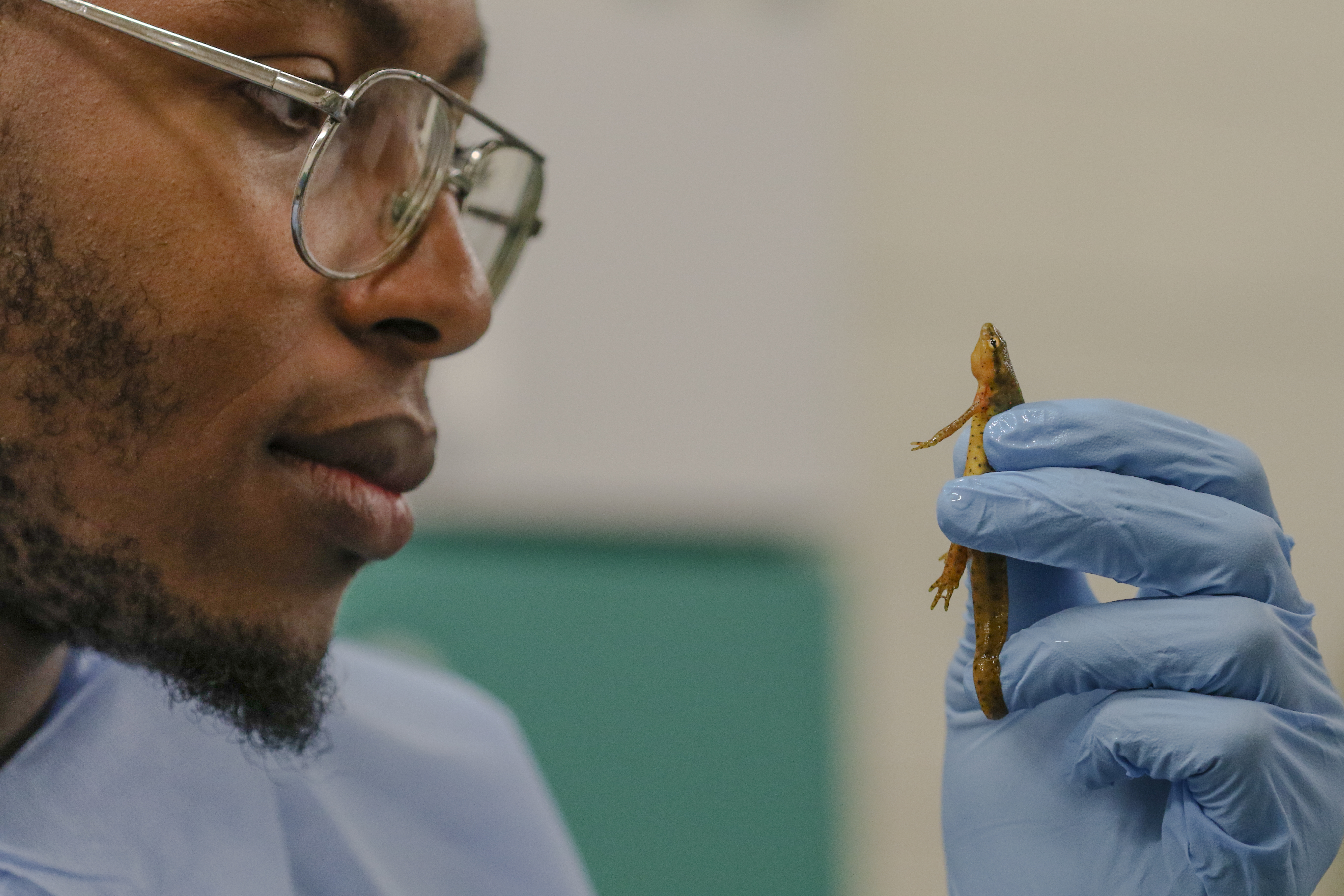

Supplement: S4 Fig — (JPG) [file pone.0235370.s004.JPG]
