# Supplementary material for: Experimental methodologies can affect pathogenicity of Batrachochytrium salamandrivorans infections
Source: PLoS One. 2020 Sep 11;15(9):e0235370. doi: 10.1371/journal.pone.0235370 (PMC7485798; doi:10.1371/journal.pone.0235370)
Supplement: Attachment — Submitted filename: Responses to Reviewer Comments (PONE-D-20-17817) Revision2.docx [file pone.0235370.s005.docx]

**Responses to Reviewer Comments**

PONE-D-20-17817

Review of Kumar et al., “Experimental methodologies can affect pathogenicity of *Batrachochytrium salamandrivorans* infections”

**NOTE: ALL LINE NUMBERS BELOW REFER TO THE TRACK CHANGES VERSION OF THE MANUSCRIPT FOR EASY REFERENCE. THE LINE NUMBERS DO NOT CORRESPOND IN THE CLEAN COPY.**

**Reviewer 1**

Reviewer #1: I commend the authors for their thorough revisions and believe the paper is much improved. My only remaining concern is that the introduction still lacks context, and as the introduction stands now the reader has no way of knowing a) why these five experimental objectives are relevant or b) what the underlying hypotheses/expectations are. I understand that these details are present in the discussion, but that is read last. An important purpose of an introduction is to establish context for the reader to understand the questions and objectives in the study before diving into the methods and results. However, I will leave it to the editor to decide whether to request this revision.

Response: We added expanded the justification for the study (Lines: 57-64), and explicitly state our hypotheses with explanations now (Lines: 94-102). NOTE: These hypotheses were *a priori* and included in early versions of the manuscript, but were removed during editing prior to submission.

**Reviewer 2**

Reviewer #2: This manuscript is much improved and with a few minor changes I think could be accepted for publication at PLOS ONE. I very much appreciate the addition of Table 1 and the additional details in the methods sections which serve to clarify the experimental design. I also think that changing the language from “tolerance/resistance” to pathogenicity is appropriate and addresses many of the initial reviewer comments. However, I do feel that some of the comments raised by reviewer 1 could be addressed more thoroughly in the text, and not simply in the rebuttal letter.

First, the supplemental figure provided for reviewer 1 could be provided as a supplemental figure to accompany the text and address many of the reviewer comments asking for more information from the Bsal qPCR results.

Response: We added the figure comparing Bsal loads at necropsy among treatments as a supplemental figure (see new Supporting Information document and the Results section for referencing Figure S1).

However I am slightly confused by one comment in the rebuttal letter that states the following “Response: We did not process all the swab samples collected as part of this experiment due to limited funds and these data weren’t necessary for inferences on pathogenicity.” This fact is not reported in the paper at all. Rather the methods section implies that all swabs were tested. Which swabs were tested and which were not? How did you test for significant differences in pathogen loads if you did not test all the swabs? This point must be clarified and transparently reported in the text.

Response: We tested for differences in Bsal loads at necropsy, which is now explicitly indicated (lines 148-149, 212,231,245, 248, 253, 256).

Additionally, this paper still fails to connect the findings to a broader significance beyond recommendations for Bsal infection experiments, which I think is a missed opportunity. First, this narrow framing is manifest in the introduction which – as initially stated by reviewer 1 – is underdeveloped. I think the second paragraph of the introduction should be a broader discussion of why some of the factors such as passage number, type of enclosure, and inoculum method would affect pathogenicity and how understanding these effects are relevant to natural systems/conservation. Then the authors can get more specific and discuss the examples they cite.

Response: We expanded the justification for the study by discussing the broader implications for two of the objectives that have logical connections to epidemiological processes (i.e., objectives 2 and 4; lines 57-64, 94-102).

At line 257 the authors could take an additional step and comment on how the infection efficiency of Bsal zoospores in water is relevant in an ecological or conservation context.

Response: We added an explanation for the ecological implications (lines 301-302).

Additionally at Line 276 the same comment applies. Discuss the tradeoffs between being able to shed your skin/shed zoospores into the environment vs the risk of being exposed to the pathogen in the water. This study does have broader implications for understating Bsal dynamics in natural settings and I think the authors should try to make some of these connections.

Response: We added an explanation for the ecological implications (lines 321-327).

Other specific notes:

The data availability should have provide a unique URL to the specific data.
Line 138 – It is unclear what you mean by “method” here. Can you specify?
Line 191 – I prefer listing the SD along with the mean.
Line 220 – Typo in this sentence “of” should be “or”

Response: The above notes were addressed (lines 166, 230, 260).
